# Electrohydrodynamic Processing of Potato Protein into Particles and Fibers

**DOI:** 10.3390/molecules25245968

**Published:** 2020-12-16

**Authors:** Ana C. Mendes, Elena Saldarini, Ioannis S. Chronakis

**Affiliations:** DTU-Food, Technical University of Denmark, Kemitorvet 202, 2800 Kgs. Lyngby, Denmark; elena.laura.saldarini@gmail.com

**Keywords:** electrospinning, electrospray, potato protein, encapsulation, vitamin release, fibers, particles

## Abstract

Potato protein particles and fibers were produced using electrohydrodynamic processing (electrospray and electrospinning). The effect of different solvents and protein concentration on the morphology of the potato protein particles and fibers was investigated. Electrosprayed particles with average diameters ranging from 0.3 to 1.4 µm could be obtained using water and mixtures of water: ethanol (9:1) and water:glycerol (9:1). Electrosprayed particles were also obtained using the solvent hexafluoro-2-propanol (HFIP) at a protein concentration of 5% *wt*/*v*. For protein concentrations above 10% *wt*/*v*, using HFIP, electrospun fibers were produced. The release of vitamin B12, as a model bioactive compound, from potato protein electrospun fibers, was also investigated, demonstrating their potential to be utilized as encapsulation and delivery systems.

## 1. Introduction

Potatoes are one of the most produced crops, and despite their low protein concentration (about 1.7%), potatoes are the second-highest protein providing crop per hectare grown after wheat [1]. Potato proteins are produced by being isolated from potato fruit juice, a byproduct of potato starch production [2]. Those proteins are often classified into three groups, namely patatins (about 40% of total soluble protein), protease inhibitors (about 50% of total soluble protein), and high-molecular-weight proteins (about 10% of total soluble protein) [1,2,3]. The patatin fraction represents a group of glycoproteins having a molecular weight ($M_{w}$) around 40 kDa and isoelectric point (pI) between pH 4.5 and 5.2 [2]. At natural pH and ambient temperature, patatin exist as a native 80 kDa dimer held together by non-covalent hydrophobic forces [2,4,5]. The protease inhibitors, the most abundant group of proteins, are a diverse group of proteins classified into seven subgroups with a molecular weight between 20 to 40 KDa and pI varying from 5 to >9 [2,5]. The other proteins in potato consist mostly of high Mw (>40 kDa), including lectins and oxidative enzymes, such as lipoxygenase, polyphenol oxidase and enzymes associated with starch synthesis [2,5].

Furthermore, potato proteins are considered as GRAS and are known for having some health benefits such as low allergenicity, antimicrobial, antioxidant and anticarcinogenic properties. In addition, potato proteins have high nutritional value because of their high digestibility and balanced amino acid composition, comparable to milk and egg proteins [6,7] and can be used as food ingredients in several diet types, including vegetarian, vegan and Pareve Kosher [7].

Despite the abundance, healthy and nutritional value of potato proteins, limited studies assess their utilization as encapsulation and delivery matrices. David et al. used potato protein to encapsulate vitamin D through nanocomplexation [7]. The nanocomplexes formed between vitamin D and the proteins proved to be efficient in protecting and reducing vitamin D losses during pasteurization and through different sets of storage conditions. In another study by Edelman et al. [8], potato protein showed great potential as a wall material for the encapsulation of lipophilic bioactives such as astaxanthin. Herein the protein demonstrated its potential to improve the solubilization, protection, and bioavailability of astaxanthin for food and dietary supplements.

Among the different technologies used to encapsulate and deliver bioactives, electrohydrodynamics (EHD, electrospinning and electrospraying) show promise as encapsulation technology, as it utilizes electrical fields that can be operated at aqueous solutions, at room temperature and without heat [9,10,11]. During the electrohydrodynamic processing [11,12], a high voltage electrostatic field charges the surface of a biopolymer solution droplet to induce the ejection of a liquid jet through a spinneret towards a grounded collector [9]. During the trajectory from spinneret to the collector, there is solvent evaporation, and if the jet elongates to produce continuous dried fibers, electrospinning is taking place [9]. For electrospray, the jet breaks down into droplets, producing dried solid nano- microparticles [10,11,12]. Parameters such as solution properties (mainly viscosity, electrical conductivity and surface tension), electrohydrodynamics conditions (applied voltage, fluid flow rate, distance from the nozzle to collector) and ambient conditions (like temperature and humidity) are critical for the production of fibers/particles [9]. Furthermore, those parameters can also be used to manipulate the size and the morphology of the electrosprayed and electrospun structures [9,10,11,12]. Encapsulation and delivery systems produced by electrohydrodynamics, allows the use of a broad range of bioactives and shell ingredients [9]. The encapsulation of sensitive compounds such as drugs [9], vaccines [13], polyphenolic antioxidants, omega-3 polyunsaturated fatty acids (PUFAs), probiotics and vitamins [10] has been successfully accomplished by electrohydrodynamics.

Vitamin B12, also known as cyanocobalamin, is an essential factor in DNA synthesis (for chromosomal replication and division) and a mediator of hematopoietic and nervous systems [14]. Consequently, vitamin B12 deficiency affects several body functions. Furthermore, this vitamin deficiency is often associated with malignant anemia patients and people with other intestinal disorders that cannot absorb this vitamin [15]. Vitamin B12 supplements are necessary, and novel delivery systems are in demand to encapsulate and facilitate the delivery process of vitamin B12. Microemulsions [16] and electrospun nanofibers of polycaprolactone [15] and chitosan/phospholipids [17] were studied for the encapsulation and delivery of vitamin B12.

This study aimed to investigate the electrospray and electrospinning processing of potato protein into particles and fibers, and to explore the potential of electrospun protein fibers as encapsulation and release matrices for vitamin B12.

## 2. Results and Discussion

### 2.1. Electrical Conductivity of the Potato Protein Solutions

Table 1 shows the values of the electrical conductivity of the potato protein samples prepared using different solvents. Overall, the protein solutions prepared in water displayed a higher electrical conductivity (7705.5 µS/cm). The addition of ethanol 10% (*v*/*v*) decreased significantly (*p* < 0.01) the conductivity of the samples comparatively to water to about 1779.2 µS/cm (Table 1). It is known that ethanol tends to decrease the conductivity of the solutions due to the non-electrolytic nature of alcohol [18]. It has been demonstrated that in mixtures of ethanol and water, the increase of ethanol concentration favors the hydrogen bonding interactions between alcohol and water, which reduces the mobility of the ions in the solution and consequently decreases the electrical conductivity of the solution [18]. Similar effect was observed with glycerol. When the protein was dissolved in water with 10% (*v*/*v*) glycerol, a statistically significant decrease in conductivity to 2958.1 µS/cm was observed. Glycerol is a solvent with higher polarity able to form water-glycerol mixtures due to the hydrogen bonding network [19].

Protein solutions prepared in HFIP displayed statistically lower conductivity (P9, 23.9 µS/cm) compared to protein solutions in water (P1 7705.5 µS/cm) (*p* < 0.01). Lower conductivity of protein solutions prepared in HFIP has also been reported in previous studies [20].

For protein solutions prepared using HFIP, the conductivity remained nearly constant with the increase of the protein concentration (Table 1). Similarly, an increase of the protein concentration from 20% *wt*/*v* to 40% *wt*/*v* using water as a solvent did not significantly change the electrical conductivity. This can be due to the higher viscosity of the solution at relatively high protein concentrations, which can limit electron mobility [21], and has been reported for gliadin [21] and zein [22] solutions.

### 2.2. Effect of Solvents on the Size and Morphology of Electrosprayed/Spun Potato Protein Particles and Fibers

#### 2.2.1. Water

**Figure 1:**
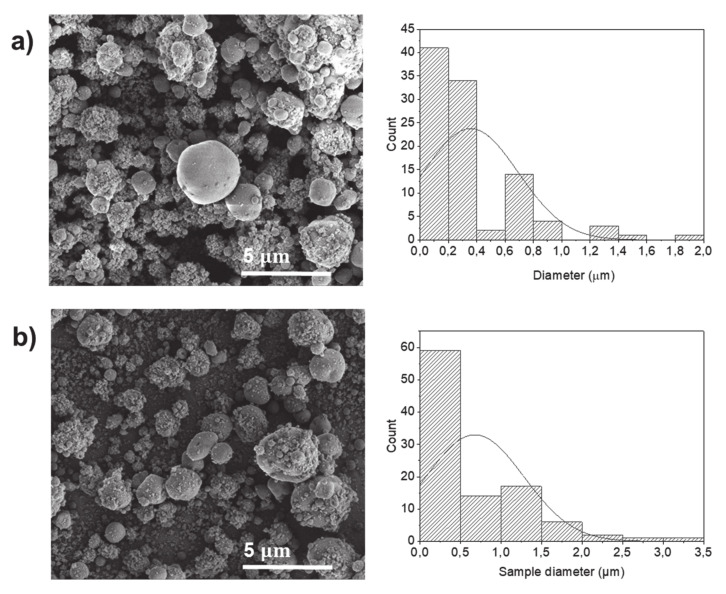
SEM images of electrosprayed potato protein particles, using water as a solvent, and respective histograms displaying their diameter distribution. Samples prepared using 20% *w*/*v* protein and processed at voltages of (**a**) 25 kV and (**b**) 30 kV.

Figure 1 shows the SEM images of the electrosprayed potato protein particles processed using a protein solution of 20% *w*/*v*. The average diameter of the potato particles was about 0.36 and 0.68 µm using voltages of 25 and 30 kV, respectively, with a heterogeneous size distribution. It is to note that protein solutions with a concentration below 20% *w*/*v* were not sufficient to produce electrosprayed particles (data not shown).

**Figure 2:**
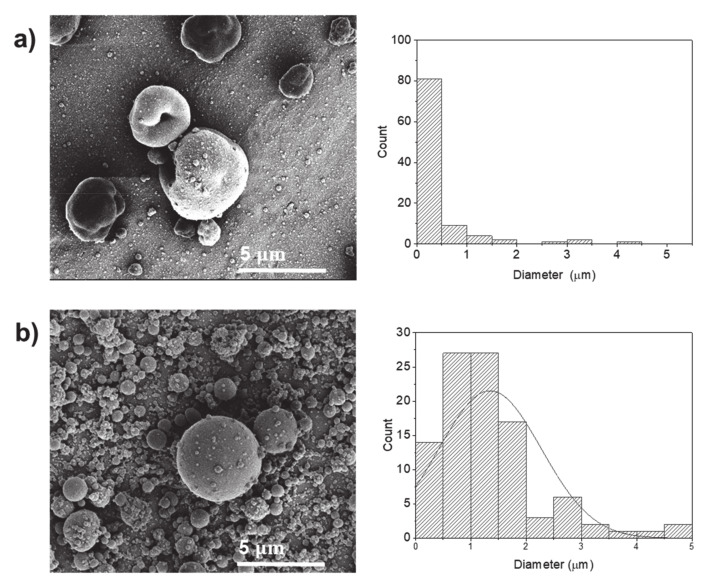
SEM images of electrosprayed potato protein particles, using water as a solvent, and respective histograms displaying their diameter distribution. Samples prepared using 40% *w*/*v* protein and processed at voltages of (**a**) 25 kV and (**b**) 30 kV.

By increasing the protein concentration to 40% *w*/*v*, the particles become more spherical in size distribution (Figure 2). Furthermore, the average size diameter of the protein particles was increased with the increase of the protein concentration, and this increase was statistically significant at the highest applied voltage (30 kV). The increase in the average diameter with the increase of the protein concentration has been observed in the electrospray processing [9,12].

The applied voltage is a critical element in the electrohydrodynamics process because it provides a surface charge to the jet and affects the diameter of the particles. However, at 20% *w*/*v* protein concentration, the increase in the voltage from 20 to 30 kV led to a significant increase in diameter size of the electrosprayed particles from 0.42 to 1.35 µm (Figure 2) due to the increase of the electrostatic stresses, which in turn, may draw more material out of the syringe [23].

#### 2.2.2. Water:Ethanol

**Figure 3:**
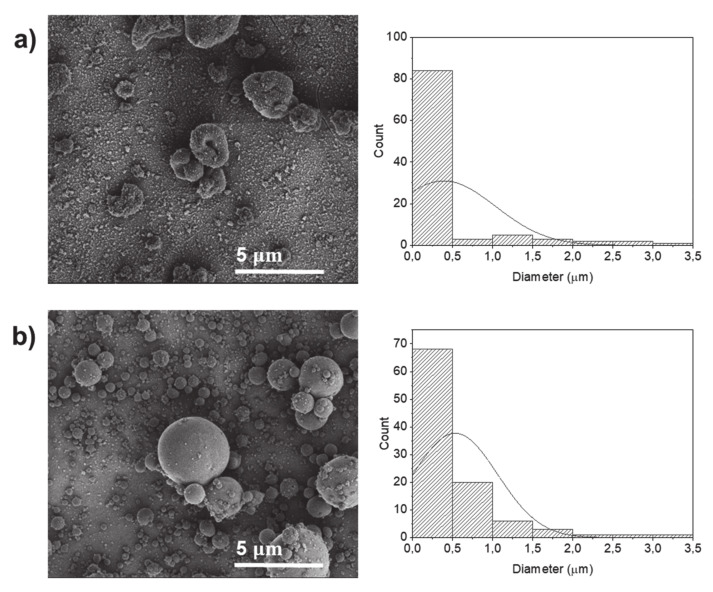
SEM images of electrosprayed potato protein particles and respective histograms displaying their diameter distribution. Samples prepared using water:ethanol (9:1) as a solvent and potato protein concentrations of (**a**) 10% *w*/*v* and (**b**) 20% *w*/*v*.

Figure 3 shows the morphology and size distribution of electrosprayed particles produced using water:ethanol (9:1) as a solvent (sample P6) using the same EHD conditions of the electrosprayed particles produced using only water as a solvent (sample P1). Overall, the addition of ethanol led to a more homogeneous size distribution and to an increase in the average size diameter of the electrospray particles from 0.36 µm to 0.53 µm (Table 1). This is probably due to the lower solubility of the potato protein with the addition of ethanol [24]. In addition, such an increase in diameter can also be related to the decrease in the conductivity of the solutions in the presence of ethanol. It has been observed that the lower conductivity of solution produces higher diameter size structures by EHD due to the shorter stretching of the electrified jet [25].

The effect of concentration on the average diameter and morphology of the electrosprayed particles using ethanol as a co-solvent was also investigated (Figure 3 and Table 1). Electrosprayed protein particles could be produced using either 10 (sample P5) or 20% *w*/*v* (sample P6) of protein. A protein concentration of 10% *w*/*v* dissolved in water:ethanol (9:1) produced particles with flattened surface and non-spherical geometries (Figure 3a) with an average diameter of 0.39 µm (Table 1). By increasing the protein concentration to 20% *w*/*v*, spherical particles were produced with higher average diameter size (0.53 µm) (Figure 3b), due to the formation of additional chain entanglements [9,12,26].

#### 2.2.3. Water:Glycerol

**Figure 4:**
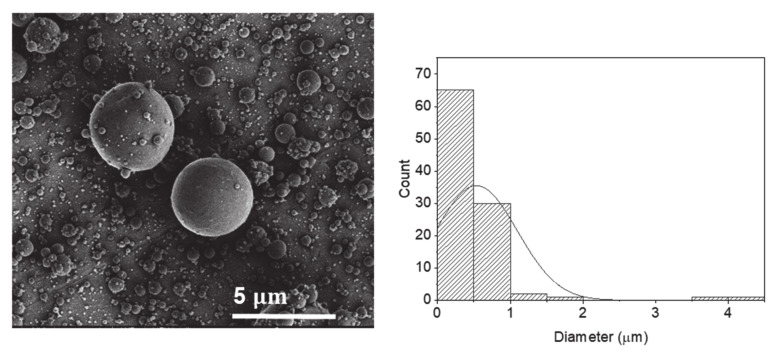
SEM images of electrosprayed potato protein particles and respective histograms displaying their diameter distribution using water:glycol (9:1) as solvents.

The effect of polar co-solvents, such as glycerol, was also investigated (Figure 4). The utilization of water:glycerol (9.1) as solvent (sample P10) created smoother and rounder electrosprayed particles (Figure 4) compared to the particles produced from aqueous solutions using the same electrospray conditions (sample P3). Furthermore, the addition of glycerol increased the average diameter of the particles from 0.42 µm to 0.53 µm (Table 1). This is in accordance with a previous study by López-Rubio et al. where an increase in size and changes in the morphology of the electrosprayed whey protein in aqueous solutions was observed by the incorporation of glycerol [27].

#### 2.2.4. Hexafluoro-2-Propanol (HFIP)

**Figure 5:**
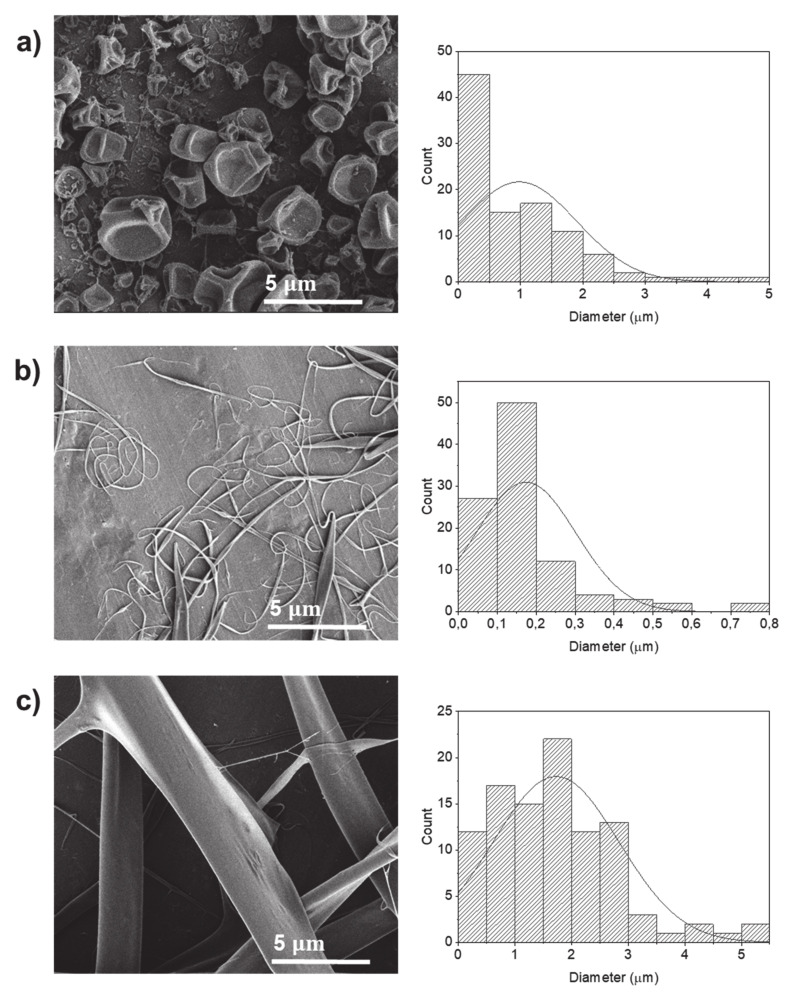
SEM images of electrosprayed potato protein particles using hexafluoro-2-propanol (HFIP) as a solvent and respective histograms displaying their diameter distribution. Samples prepared at potato protein concentrations of (**a**) 5% *w*/*v*, (**b**) 10% *w*/*v* and (**c**) 20% *w*/*v*.

Electrosprayed particles could be produced at a protein concentration of 5% *w*/*v* dissolved in HFIP (sample P7) with an average size diameter of 0.98 µm (Figure 5a). This concentration is much lower in comparison to the concentration of 20% *w*/*v* required to produce electrospray particles using water as a solvent (Figure 1). Electrosprayed particles prepared using HFIP displayed a flattened morphology with a dimpled structure, which may be related to the incomplete evaporation of the solvent [28] during their trajectory from the tip of the needle to the collector.

By increasing the protein concentration to 10% *w*/*v* (sample P8), electrospun protein fibers with average diameters of 0.17 µm were produced. At the protein concentration of 20% *w*/*v*, fibers with flattened surface and average diameter of 1.72 µm were produced (Figure 5 and Table 1). A similar effect of the formation of particles and fibers depending on the protein concentration using HFIP as solvent was also obtained for the EHD processing of fish sarcoplasmic proteins [29] and electrospun amaranth fibers [20]. HFIP is known as a solvent that favors the formation of random coil structures with a subsequent increase in the hydrodynamic volume and the degree of biopolymer entanglements in solution [20]. Moreover, an increase in the protein concentration led to an increase of the molecular entanglements and the subsequent fluid extension to form fibers instead of particles due to the Rayleigh instability [21]. Furthermore, fibers could also be obtained by the breaking and re-forming of disulfide bonds that occur via thiol/disulfide interchange reaction with solvents such as HFIP [21]. It is also to note that 20% *w*/*v* of potato protein dissolved in aqueous solution produced particles (Figure 1b), while dissolved in HFIP produced fibers instead (Figure 5c).

### 2.3. Encapsulation and Release of Vitamin B12 from Potato Protein Fibers

**Figure 6:**
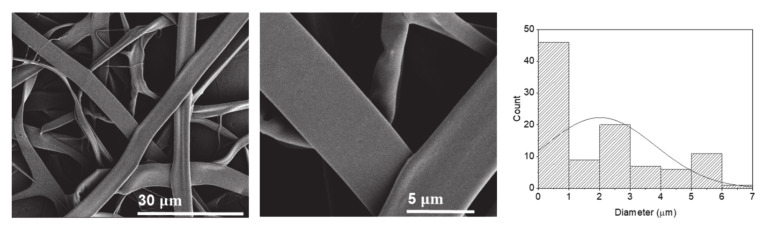
SEM images of electrospun potato protein fibers loaded with 0.05% *w*/*v* vitamin B12 and respective histogram displaying their diameter distribution.

Vitamin B12 was used as a model compound to investigate the potential of the electrospun potato protein fibers to encapsulate and release bioactives. SEM images of the potato protein fibers using HFIP as solvent loaded with vitamin B12 are displayed in Figure 6. Morphologically, the electrospun fibers did not change with the inclusion of the vitamin (Figure 6).

Although fibers maintained their flattened morphology, their average diameter increased slightly from 1.72 µm to 2.01 µm (Table 1).

Potato protein fibers were found to be insoluble when they were immersed in an aqueous PBS buffer solution. The fact that the water-soluble potato protein became insoluble after electrospinning may be due to the proteins being in an unfolded state when turned into fibers. Unfolding may be caused either by the solvent or by the electrospinning process and has been observed previously for electrospun fish sarcoplasmic proteins nanofibers produced with HFIP as solvent [29].

**Figure 7:**
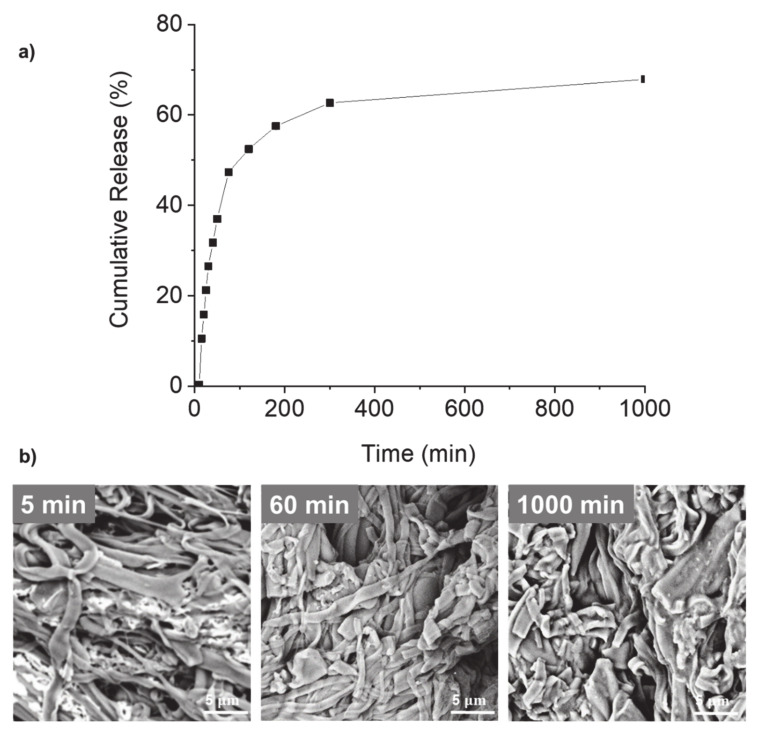
(**a**) Cumulative release of vitamin B12 from electrospun potato protein fibers immersed in phosphate-buffered saline (PBS) solution (pH = 7.4, 37 °C). (**b**) SEM images of the electrospun potato protein fibers after their immersion in PBS (37 °C) for 5 min, 60 min and 1000 min (*n* = 3).

The release profile of vitamin B12 from potato protein electrospun fibers immersed within PBS buffer solution is exhibited in Figure 7. An initial burst release profile where about 50% of the vitamin was released was observed within the first 60 min. Between 75 to 300 min, a slight increase in the cumulative release of vitamin B12 was observed up to 63.3%. Beyond that and up to 1000 min, no significant changes in the cumulative release of vitamin B12 were detected, and a steady state was reached with 68% released vitamin. Previous studies have also shown the release of an encapsulated compound from other insoluble fibers, and the maximum release was explained to depend on the surface properties (e.g., porosity) of the fibers [30,31,32]. They also showed that the release was facilitated by a two-step mechanism: a rate-limiting step being desorption of the compound from the nanoporous surface of the fibers, followed by diffusion into the buffer. In another study, the release of vitamin B12 from hydrophobic polycaprolactone electrospun fibers was low (34%), in comparison to the release from plasma-treated hydrophilic fibers of 95% [15] also suggesting that the surface properties of the fibers modulate the maximum release of the bioactive.

Moreover, the release of vitamin B12 from electrospun potato protein fibers was analyzed using the Korsmeyer–Peppas model [33]. This model estimates whether the release of the compound from a matrix follows Fickian diffusion, which can be predicted through the calculation of the coefficient “n” estimated from linear regression of the log(Cumulative Release) as a function of log(Time). The coefficient “n” was 0.91, suggesting that the release mechanism did not follow a Fickian diffusion, and vitamin B12 was probably released due to changes on the surface of the potato protein fibers.

The morphology of the potato protein fibers loaded with B12 after their immersion in PBS buffer for 5 to 1000 min is shown in Figure 7b. Swelling of the matrix with a consequent slight increase in average diameter over time was observed. However, their fibrillar structure remained intact due to the insolubility of the fibers in an aqueous buffer solution as a result of protein conformational changes when dissolved in HFIP [21].

## 3. Materials and Methods

### 3.1. Materials

The potato protein (PK15) was kindly donated by KMC (Brande, Denmark) and used as received. Potato protein solutions were prepared, dissolving the protein in various solvents: MilliQ water, ethanol (VWR chemicals, Paris, France), glycerol (Scharlau Chemie, Barcelona, Spain), and 1,1,1,3,3,3-hexafluoro-2-propanol-2,2 (HFIP) (Sigma-Aldrich, Shanghai, China). The encapsulation of vitamin B12 (Sigma-Aldrich, Saint Louis, MO, USA) was done by adding 0.05% *w*/*v* of vitamin B12 to sample P9. The protein concentration and solvents used are described in Table 2.

### 3.2. Conductivity of the Solutions

The conductivity of each solution was determined using a conductivity meter (Thermos Scientific, Orion Versa Star Pro, Waltham, MA, USA). The conductivity cell (Thermos Scientific, Orion 013005MD, Waltham, MA, USA) was dipped into 10 mL of potato protein solutions. Two measurements of each solution were recorded, and tests were conducted at room temperature (23 °C).

### 3.3. Electrospray and Electrospinning

The EHD system consisted of a high-voltage generator (ES50P-10W, Gamma High Voltage Research, Inc., Ormond Beach, FL, USA). A syringe pump (New Era Pump Systems, Inc., Farmingdale, NY, USA) was used to feed the protein solutions at a flow rate of 0.02 or 0.06 mL/min using 24 and 19G needles (0.559 mm and 1.067 mm diameter, respectively). The distance between the needle and the collector was 15 cm, and the applied voltage was in the range of 25–30 kV. The electrospray/spinning system was placed inside a chamber, and the temperature and relative humidity were 23 °C and 32%, respectively. The different EHD conditions used for each sample are described in Table 2. The collector was a grounded squared metal plate, covered with aluminum foil, where the particles and fibers were collected and left to dry overnight in a fume-hood.

### 3.4. Scanning Electron Microscopy (SEM)

For morphology and size analysis, samples were attached on metal stubs with double-sided adhesive carbon tape and sputter-coated with 6 nm of gold (Leica Coater ACE 200, Leica, Vienna, Austria) prior to their imaging in a Quanta FEG 3D (FEI, Eindhoven, The Netherlands) scanning electron microscope. Visualization of the samples after the release studies was conducted using a Phenom Pro scanning electron microscope (Thermo Fisher Scientific, Eindhoven, The Netherlands). Particles and fibers diameters were measured using the image visualization software ImageJ (National Institutes of Health, Bethesda, MD, USA). The average diameters and diameter distributions were determined by measuring 100 particles or fibers.

### 3.5. Release of Vitamin B12

The release of vitamin B12 from electrospun potato protein fibers was investigated in phosphate-buffered saline (PBS) solution (0,01 M, pH 7.4). Fibers (3 mg) were placed into 1.5 mL of PBS in a thermos shaking water-bath (100 rpm at 37 °C) (Julabo SW22, Seelbach, Germany). Supernatant aliquots (100 µL) samples were withdrawn at the time intervals of 5, 10, 15, 20, 25, 30, 40, 50, 75, 120, 180, 300, 1000 min, vortexed (Heidolph REAX 2000, Schwabach, Germany), centrifuged for 2 min at 13,000 rpm (Centrifuge 5424, Eppendorf, assembled in the USA) and replaced with the same volume of fresh PBS. The release of vitamin B12 was analyzed by determining the absorbance at 365 nm with ultraviolet-visible spectrophotometer NanoDrop One^c^ (Thermo Scientific, Roskilde, Denmark). All the release tests were carried out in triplicate.

### 3.6. Statistical Analysis

Tukey’s test for multiple comparison test (*p ≤ 0.05) was employed using Origin Pro 2019 (9.6.0.172) software (OriginLab, Northampton, MA, USA) to assess the statistical differences between the different conditions.

## 4. Conclusions

Potato protein particles and fibers were developed using electrohydrodynamics processing. The morphology of the produced structures was mostly affected by the solvent and the concentration of the protein used. Electrosprayed particles with average diameters ranging from 0.3 to 1.4 µm could be obtained using water and water:ethanol (9:1), water:glycerol (9:1) and HFIP. Fiber structures were obtained only when HFIP was used as solvent. Potato protein electrospun fibers swell and remain insoluble when they were immersed in PBS buffer solution at 37 °C and can be exploited to encapsulate and release bioactive compounds such as the hydrophilic vitamin B12. Nevertheless, further studies are needed in order to assess the delivery properties, cytotoxicity and bioavailability of vitamin B12 using potato protein structures developed by EHD processing.

## Figures and Tables

**Table 1:** Electrical conductivity of the potato protein solutions and the average diameter of the particles and fibers produced by electrohydrodynamics.

Sample Designation	Electrical Conductivity (µS/cm)	Average Diameter (µm)
P1	7705.5 ± 146.4	0.36 ± 0.34 (particles)
P2	7705.5 ± 146.4	0.68 ± 0.60 (particles)
P3	7651.5 ± 61.4	0.42 ± 0.71 (particles)
P4	7651.5 ± 61.4	1.35 ± 0.93 (particles)
P5	1263.7 ± 35.8	0.39 ± 0.64 (particles)
P6	1779.2 ± 1.4	0.53 ± 0.52 (particles)
P7	20.3 ± 0.2	0.98 ± 0.92 (particles)
P8	20.6 ± 0.7	0.17 ± 0.13 (fibers)
P9	23.9 ± 1.2	1.72 ± 1.11 (fibers)
P10	2958.1 ± 6.8	0.53 ± 0.56 (particles)
P11	24.5 ± 0.9	2.01 ± 1.79 (fibers)

**Table 2:** Potato protein solutions and electrohydrodynamics processing parameters used.

Sample Designation	Concentration PK15 (% *w*/*v*)	Solvent (% *v*/*v*)	Flow Rate (mL/min)	Needle (G)	Distance * (cm)	Voltage (kV)
P1	20	H_2_O	0.02	24	15	25
P2	20	H_2_O	0.02	24	15	30
P3	40	H_2_O	0.02	19	15	25
P4	40	H_2_O	0.02	19	15	30
P5	10	H_2_O:ethanol (9:1)	0.02	24	15	25
P6	20	H_2_O:ethanol (9:1)	0.02	24	15	25
P7	5	HFIP	0.02	24	15	25
P8	10	HFIP	0.02	24	15	25
P9	20	HFIP	0.02	24	15	25
P10	40	H_2_O: glycerol (9:1)	0.02	19	15	25
P11 **	20	HFIP	0.02	24	15	25

* Distance between the spinneret needle to the collector. ** 0.05% *w*/*v* of vitamin B12.
